# The sinking dynamics of a solid intruder in concentrated cornstarch suspensions studied using ultra-fast magnetic resonance imaging

**DOI:** 10.1039/d6sm00385k

**Published:** 2026-05-15

**Authors:** Nikolay K. Kirov, Christopher P. McLaren, Klaas P. Pruessmann, Christoph R. Müller, Alexander Penn

**Affiliations:** a Institut de Mécanique des Fluides de Toulouse, IMFT, Université de Toulouse, CNRS Toulouse France kirov.nikolay@imft.fr +33 789 32 44 11; b Department of Mechanical and Process Engineering, ETH Zürich 8092 Zürich Switzerland; c Institute for Biomedical Engineering, ETH Zürich and University of Zürich 8092 Zürich Switzerland; d Institute of Process Imaging, Hamburg University of Technology 21073 Hamburg Germany alexander.penn@tuhh.de +49 40 30601 3801

## Abstract

The sinking of intruders in concentrated cornstarch suspensions is governed by localized, transient dynamics that are difficult to access experimentally because of the opacity of the material. Here, we use real-time magnetic resonance imaging (MRI) to investigate the sinking of spherical intruders in cornstarch suspensions at solid fractions *ϕ*_0_ = 0.41 and 0.44. Ultra-fast 1D MRI measurements show that, consistent with earlier reports, the intruders pass through an impact transient, an oscillatory sinking regime, and late stop and go cycles near the bottom boundary. By varying the intruder diameter at fixed suspension composition, we find that larger intruders sink more slowly, while the oscillations in the intermediate sinking regime exhibit similar characteristic frequencies and amplitudes for all three sizes. A reduced drag-memory model further shows that these oscillatory sinking velocities can be described reasonably well by a common phenomenological history-dependent response for the presented conditions. The 1D MRI signal maps reveal synchronous signal modulations around the intruder, indicating that the oscillatory motion is coupled to repeated growth and partial relaxation of a perturbed suspension region. Complementary 2D MR velocimetry shows that the motion is not purely vertical, but also includes oscillations in the horizontal direction. We show that the surrounding flow and strain rate fields are strongly heterogeneous, with deformation becoming progressively concentrated beneath the intruder prior to arrest.

## Abstract 2

Real-time MRI links the oscillatory motion of sinking intruders in cornstarch suspensions to the suspension response. Evolving perturbed regions and shear rate heterogeneities around the intruder indicate coupled, history-dependent dynamics.

## Graphical abstract

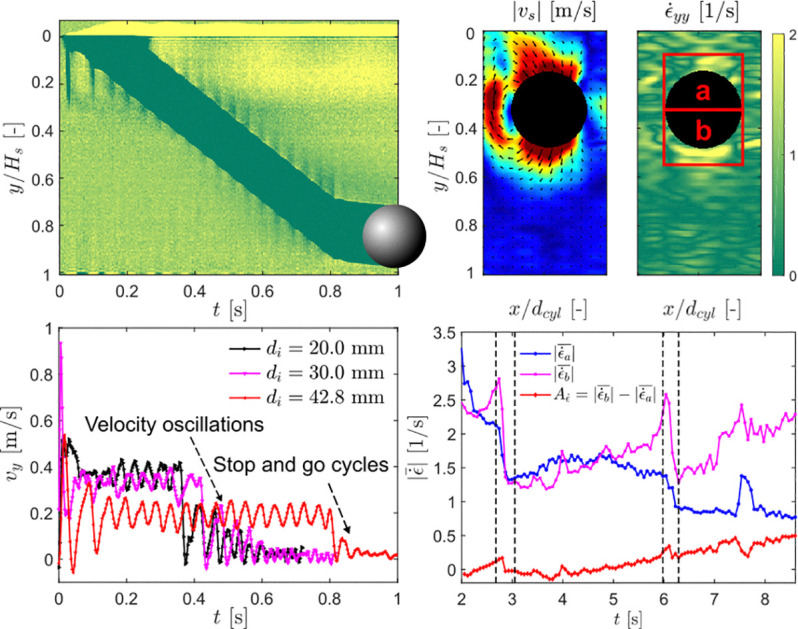

## 1 Introduction

Suspensions, *i.e.*, mixtures of solid particles dispersed within a liquid, are prevalent in both natural settings, such as landslides, muddy water, and blood, and in industrial applications, including cosmetics, pharmaceuticals, coatings, and food products.^1,2^ Under forcing, suspensions can display shear thinning, in which the apparent viscosity, *η*, decreases as the material is sheared more strongly,^3–5^ as well as shear thickening, in which the apparent viscosity instead increases with forcing.^6–10^ In sufficiently concentrated systems, this growth in resistance can become so pronounced that the suspension dynamically jams into a transient solid-like state capable of supporting loads.^11–15^ Understanding these responses is important not only from a fundamental point of view, but also for understanding and improving technologies and processes involving suspensions, as well as for applications that directly exploit shear thickening behavior, such as shock absorption, impact mitigation, and protective materials.^16–18^

A central advance of the last decade has been the emergence of a unified framework for shear thickening in dense non-Brownian suspensions.^19–24^ In the Wyart–Cates theory, particle contacts are lubricated at low stress because short-range repulsive forces and the intervening fluid keep neighboring particles apart. Once the applied stress becomes large enough to overcome this stabilizing repulsion, an increasing fraction of particle contacts becomes frictional.^25,26^ This stress-activated transition lowers the jamming solid fraction of the suspension from its lubricated value toward a smaller frictional one.^20,21^ Because the viscosity rises steeply as the actual solid fraction approaches the relevant jamming fraction, this shift produces shear thickening.^27,28^ The framework then distinguishes several regimes. At relatively low solid fraction, the viscosity increases smoothly with forcing, giving continuous shear thickening (CST). At larger solid fraction, the increase becomes abrupt, leading to discontinuous shear thickening (DST), *i.e.*, a sharp jump between a low-viscosity lubricated state and a high-viscosity frictional state. At still larger solid fraction, the high-stress frictional state is itself jammed, so the suspension can no longer sustain homogeneous flow without dilation (*i.e.*, an increase in volume as the particles move apart under shear); this regime is referred to as shear jamming (SJ).^29–31^ In this sense, stress activates frictional contacts, the solid fraction sets the distance to jamming, and confinement converts dilatancy into large normal stresses and load-bearing contact networks. Direct support for this frictional scenario has been obtained experimentally down to the scale of pairwise interactions.^32^

Cornstarch-water suspensions are the prototypical example of this class of materials because they display particularly dramatic DST, impact-activated solidification, and shear jamming behavior.^13,29,33^ Cornstarch particles are polydisperse,^34^ irregular in shape,^35^ porous,^36^ and the rheology is highly sensitive to solvent composition and ambient humidity.^37,38^ In addition, cornstarch exhibits a low-stress yield-like response^1,35^ and strong flow heterogeneities that are not fully captured by the simplest homogeneous Wyart–Cates description.^39,40^ Recent work has further suggested that cornstarch cannot be understood as a purely repulsive hard-particle suspension, since adhesive or attractive interparticle forces may contribute to its rheology.^40^ Cornstarch is therefore best viewed as a material that broadly follows the modern frictional-jamming framework while retaining additional physicochemical complexity specific to the particles and solvent.

This distinction is important because, in cornstarch, macroscopic DST does not simply correspond to a local jump onto a homogeneous high-viscosity steady branch. Using combined rheometry and MRI, Fall *et al.*^39^ showed that DST is instead associated with flow localization and the coexistence of flowing and jammed regions generated by shear-induced dilation under confinement; within the flowing regions, the local rheology does not necessarily exhibit discontinuous shear thickening and may even remain shear thinning. Subsequent studies have reinforced this picture by showing that thickening in cornstarch suspensions is intrinsically heterogeneous and often unsteady, involving propagating density waves carrying large normal stresses,^41^ localized stress fluctuations,^42^ and particle migration.^43^

A line of research closely related to the present study examined the motion of impacting and otherwise driven intruders in concentrated cornstarch suspensions. In particular, Waitukaitis and Jaeger showed that an impacting intruder generates a dynamic jamming front that propagates ahead of it and rapidly transforms an initially fluid-like suspension into a transient solid capable of sustaining very large stresses.^13,44^ The front speed at impact satisfies1
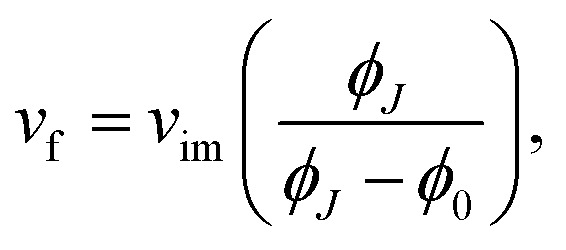
where *v*_im_ is the impact speed, *v*_f_ the front speed, *ϕ*_0_ the stress-free solid fraction, and *ϕ*_*J*_ the solid fraction for isotropic jamming.^44^ These results showed that solidification in dense suspensions need not arise from steady homogeneous shear, but can instead result from the rapid growth of a jammed region and the associated momentum transfer.^13^ Later experiments further showed that the front propagates both axially and transversely, with an approximately constant axial-to-transverse speed ratio (measured as ≈2 in a quasi-2D configuration),^45^ and that these fronts are more precisely understood as dynamic shear jamming fronts governed by accumulated shear and anisotropic stress transmission rather than by large densification.^15,29^ Related driven-suspension experiments have also revealed unsteady drag responses and self-sustained velocity oscillations, for example, for a torque-driven rotating cylinder in a shear thickening suspension.^46^ Together with dynamic jamming phenomena explored in boundary-driven shear,^47^ under extensional forcing,^48^ and around towed intruders in dense suspensions,^49^ these studies provide a natural framework for interpreting intruder motion in cornstarch suspensions in terms of front propagation, stress transmission, dynamic shear jamming, and history-dependent drag.

Specifically for sinking intruders, von Kann *et al.*^50,51^ extended the study of cornstarch suspensions beyond the initial impact zone by tracking the vertical motion of a marked wire attached to an intruder. They found that during descent through the bulk of the suspension, the intruder velocity does not evolve monotonically but instead exhibits persistent oscillations, while near the bottom of the container the motion undergoes a series of stop and go cycles. Motivated by the work of Deegan on persistent holes in particulate suspensions,^52^ the bulk velocity oscillations were rationalized using a phenomenological hysteretic model, but this description required oscillation parameters, such as the measured amplitudes and frequencies, to be supplied from the experiments themselves, leaving it largely non-predictive and lacking a physical link to the suspension dynamics.^51^ To account for the stop and go motion near the bottom of the container, they further introduced a minimal jamming model based on a force balance on the sinking intruder,2
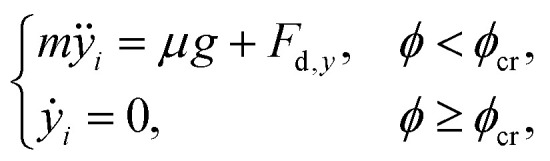
together with an evolution equation of the solid fraction:3
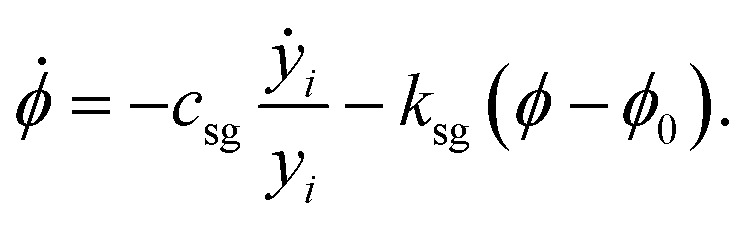


In their model, *m* = *m*_*i*_ + *m*_add_ is the total mass, with *m*_*i*_ the intruder mass and *m*_add_ = *ρ*_s_*V*_*i*_/2 the added mass associated with the displaced suspension, where *ρ*_s_ is the suspension density and *V*_*i*_ the intruder volume; *y*_*i*_ is the vertical position of the intruder, *μ* = *m*_*i*_ − *ρ*_s_*V*_*i*_ its buoyancy-corrected mass, *ϕ*_cr_ a critical solid fraction, *c*_sg_ a dimensionless phenomenological constant associated with compression, and *k*_sg_ the relaxation rate.^51^

Because the experiments of von Kann *et al.*^50,51^ accessed essentially only the intruder trajectory through an external marker, the spatiotemporal evolution of the surrounding suspension could not be observed directly. In the present work, we use ultra-fast magnetic resonance imaging (MRI) to study the sinking motion of spherical intruders in concentrated cornstarch-water suspensions. Real-time MRI^53^ provides non-invasive access to the intruder position, the surrounding velocity field, and spatial variations in MRI signal intensity throughout the descent. In contrast to earlier optical wire-tracking measurements,^50,51^ this approach allows us to relate the intruder kinematics directly to the evolving state of the surrounding opaque suspension. This is important for identifying the suspension response associated with the oscillatory sinking regime and, in turn, for clarifying the physical mechanisms underlying these oscillations. Moreover, the 2D MR velocimetry measurements allow us to examine how the surrounding flow field and deformation become redistributed as the intruder evolves from the oscillatory sinking regime into the stop and go regime, thereby providing a suspension-based perspective on the transitions between these distinct modes of motion.

The article is organized as follows. Section 2 describes the experimental methods. Section 3 presents and discusses the results, beginning with one-dimensional MRI measurements and followed by two-dimensional MR velocimetry. Finally, Section 4 summarizes the main conclusions.

## 2 Methods

**Fig. 1:**
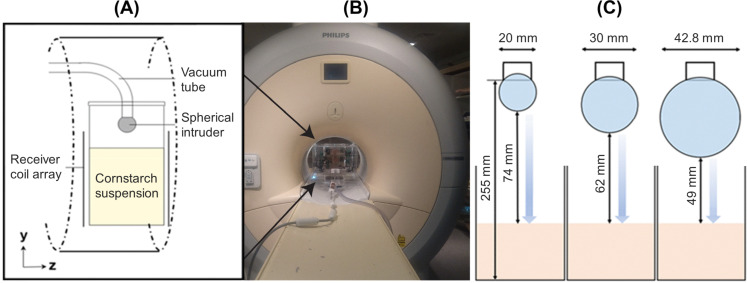
Experimental configuration. From left to right: (A) schematic illustration of the RF receiver array positioned around the cylinder containing the cornstarch suspension, together with the initial position of the intruder before release; (B) photograph of the experimental setup inside the bore of the MRI scanner; and (C) spherical intruder diameters, *d*_*i*_, and fall heights, *H*_fall_.

In this work, we utilized a 3 T medical MRI scanner (Achieva® 3T, Philips Healthcare, The Netherlands). A cylindrical polymethylmethacrylate (PMMA) container, with an internal diameter of *d*_cyl_ = 84 mm and a height of *H*_cyl_ = 270 mm, was used to contain the cornstarch suspension. A custom-designed 16-channel radiofrequency (RF) receiver coil array was positioned around the container, as shown in Fig. 1A and B. The cornstarch suspension was then poured into the container up to a height of *H*_s_ = 165 mm.

Zirconia (ZrO_2_) spheres with a density of *ρ*_*i*_ = 5680 kg m^−3^ and diameters of *d*_*i*_ = 20 mm, 30 mm, and 42.8 mm were used as intruders. The top of each sphere was initially held at a height of 255 mm above the container bottom by means of a PVC tube connected to a vacuum pump (see Fig. 1C). The applied vacuum was sufficient to keep the intruder in place. Release was achieved by switching off the vacuum and was manually synchronized with the start of the MRI data acquisition.

To estimate the impact velocity of the intruders, free-fall kinematics were used, neglecting the influence of the surrounding air, *i.e.*, 
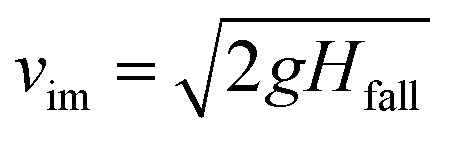
, where *g* is the gravitational acceleration and *H*_fall_ is the free-fall height. The estimated impact velocities were 1.2 ms^−1^, 1.1 ms^−1^, and 0.98 ms^−1^ for spheres of diameter 20 mm, 30 mm and 42.8 mm, respectively. Previous findings by Oyarte Gálvez *et al.*^37^ suggest that, while the magnitude of the drop height affects the initial impact response, it does not alter the subsequent settling behavior of intruders in cornstarch suspensions. Since the present work primarily focuses on the settling behavior, the experimental configuration was not modified to account for the slight differences in the free-fall heights between the different intruder diameters.

### 2.1 Ultra-fast 1D MRI profile measurements

**Fig. 2:**
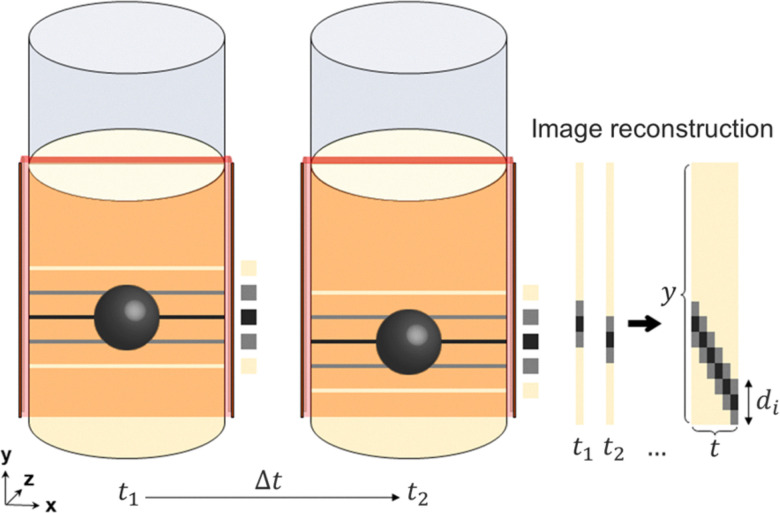
Schematic illustrating the information obtained from the 1D MRI signal intensity profiles. In these measurements, a central slice of thickness 10 mm in the *xy*-plane (red) is excited. The resulting pixel series represents the horizontally averaged MRI signal intensity, with black indicating low signal intensity, *i.e.* the intruder, and beige indicating high signal intensity, *i.e.* the suspension.

Ultra-fast 1D MRI profile measurements were conducted to track the vertical sinking of the intruders. These measurements, performed at high temporal (Δ*t* = 2.5 ms) and 1D spatial resolution (Δ*y* = 0.4 mm), enable the tracking of the complex sinking dynamics of the intruders and the derivation of their sinking velocity and acceleration. A schematic illustrating the information obtained from the 1D spin density profiles is provided in Fig. 2.

Each profile presents a vertically resolved intensity signal obtained from horizontally averaged MRI data of the excited slice (marked in red). The resulting time series yields a temporally resolved position map of the water-free and thus MRI-invisible intruder.

Table 1 summarizes the key parameters of the 1D MRI sequence. In the Philips protocol, the 1D measurements were implemented as a dynamic fast field echo sequence, corresponding to a T1FFE/FLASH-type gradient-echo acquisition. The sequence used a repetition time of 2.5 ms, an echo time of 1.14 ms, and a flip angle of 15°, with no velocity encoding, diffusion weighting, or flow compensation applied. Although this sequence was not specifically designed to enhance sensitivity to coherent or incoherent motion, the combination of a short repetition time, repeated 15° excitations, and absence of flow compensation can make the measured magnitude signal sensitive to suspension motion within the excited slice.^54^

**Table 1:** Main acquisition parameters of the MRI sequences used in this study

Imaging parameter	1D MRI	2D MR velocimetry
MRI pulse sequence	Gradient echo (FLASH)	Velocity-encoded echo planar imaging
Field of view [*x*, *y*, *z*]	[125 mm, 200 mm, 10 mm]	[125 mm, 200 mm, 10 mm]
Spatial resolution [Δ*x*, Δ*y*]	[—, 0.4 mm]	[5.7 mm, 3.1 mm]
Temporal resolution	2.5 ms	62 ms
Echo time	1.1 ms	4.7 ms
Repetition time	2.5 ms	10.3 ms
Flip angle	15°	40°
Encoded velocity [*v*_enc,*x*_, *v*_enc,*y*_]	—	[0.15 ms^−1^, 0.15 ms^−1^]

As a sensitivity test, the repetition time of the pulse sequence was varied to assess whether intruder dynamics, such as oscillations during sinking, are influenced by the MRI acquisition. No measurable change in the recorded sinking behavior was observed.

### 2.2 2D phase-contrast MR velocimetry measurements

Additional 2D MR velocimetry was employed to measure the flow field of the suspension around the sinking intruder. A velocity-encoded echo-planar imaging (EPI) pulse sequence with bipolar magnetic field gradients was used for this purpose.^53^ Measurements were acquired in the central, 10 mm-thick vertical (*xy*) slice. The MR velocimetry experiments had a temporal resolution of 62 ms and a spatial resolution of 5.7 mm × 3.1 mm, using velocity encoding with *v*_enc_ = 0.15 ms^−1^ in both the *x* and *y* directions. The resolution of the scans was interpolated onto a 2 mm × 2 mm grid using zero filling before analyzing the velocities and calculating the strain rates.

These MR velocimetry experiments not only determined the suspension velocity, yielding 2D maps of *v*_s,*x*_ and *v*_s,*y*_ in the *x* and *y* directions, respectively, but also captured the *xy* position of the intruder, allowing us to correlate the motions of the intruder and the suspension. The key parameters of the velocity-encoded EPI pulse sequence are also summarized in Table 1.

### 2.3 Preparation of the suspension

In the experiments reported here, concentrated suspensions of cornstarch (Coop, Switzerland) and deionized water, with solid fractions (by volume) of *ϕ*_0_ = 0.41 and *ϕ*_0_ = 0.44, were prepared. To reduce the longitudinal relaxation time of the water, *T*_1_, to approximately 60 ms, 3.25 ml of the gadolinium-based MR contrast agent Gadovist® (1 mmol ml^−1^, Bayer Healthcare, Germany) was added per liter of water. This reduction in *T*_1_ enabled faster repetition of the MRI sequence and, consequently, improved the temporal resolution. At this low concentration, the contrast agent did not measurably affect the rheological behavior of the suspension.

In contrast to common practice in cornstarch suspension experiments, no CsCl was added to density-match the liquid phase to the particles. Such additives were avoided here because they produced pronounced bright MRI signal contributions that compromised image contrast and hindered reliable tracking of the intruder motion. To minimize sedimentation effects, the non-density-matched cornstarch suspension was mixed thoroughly immediately before each experiment.

## 3 Results and discussion

In this section, we present the experimental results obtained from MRI measurements of intruders sinking in cornstarch suspensions. We first analyze the intruder dynamics extracted from ultra-fast 1D MRI profiles. We then present complementary observations from 2D MR velocimetry, which provide spatially resolved information on the suspension flow field around the intruder. We note, however, that the 1D MRI and 2D MRI measurements were performed at different solid fractions, namely *ϕ*_0_ = 0.41 and *ϕ*_0_ = 0.44, respectively.

### 3.1 Insights from ultra-fast 1D MRI profile measurements

**Fig. 3:**
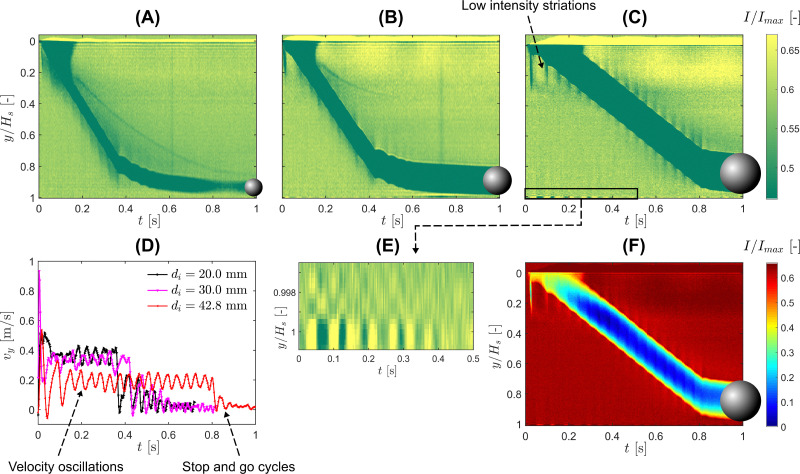
Sinking of spherical intruders of different diameters, *d*_*i*_, and constant density *ρ*_*i*_ = 5680 kg m^−3^ in a cornstarch suspension with solid fraction *ϕ*_0_ = 0.41, tracked by ultra-fast 1D MRI. The top three panels show the acquired intensity profiles for intruders of diameter (A) *d*_*i*_ = 20 mm, (B) *d*_*i*_ = 30 mm, and (C) *d*_*i*_ = 42.8 mm. Panel (D) shows the corresponding sinking velocities as a function of time, revealing clear oscillations as well as the occurrence of stop and go cycles. Panel (E) provides a close-up of the intensity signal near the bottom of the container, while panel (F) shows an enhanced-contrast color map for the experiment with the 42.8 mm intruder.

Fig. 3A–C shows spatiotemporal 1D MRI profiles for zirconia intruders of diameters 20, 30, and 42.8 mm, each with density 5680 kg m^−3^. Because the intruders were MRI-invisible, they appeared as regions of reduced signal intensity moving downward along the vertical coordinate as they sank through the suspension. The intruder position, *y*_*i*_(*t*), was extracted from these profiles using a normalized cross-correlation algorithm with a reference intruder template. The instantaneous sinking velocity, *v*_*y*_(*t*) = d*y*_*i*_/d*t*, was then computed from the smoothed trajectories (see Fig. 3D).

#### 3.1.1 Intruder motion

It can be seen that, following an initial transient at impact, the intruders enter a quasi-steady sinking regime within approximately 0.05–0.1 s. Superimposed on the mean descent are persistent velocity oscillations, which remain visible throughout the bulk sinking process. As the intruder approaches the bottom of the container, these are followed by clear stop and go cycles, consistent with the marked-wire tracking observations of von Kann *et al.*^51^ Here, by varying the intruder size within the same suspension, we show that such oscillations are observed for freely sinking, laterally unconstrained intruders across the full size range investigated. The corresponding mean sinking velocities are approximately 0.37 ms^−1^, 0.33 ms^−1^, and 0.19 ms^−1^ for *d*_*i*_ = 20, 30, and 42.8 mm, respectively, indicating that the mean sinking velocity decreases as the intruder size increases. This trend contrasts with Stokes settling in a Newtonian fluid, for which the settling velocity scales as *v*_*y*_ ∼ *d*_*i*_^2^/*η*. The observed decrease in the mean sinking velocity with increasing intruder diameter is therefore most likely attributed to the shear thickening character of the cornstarch suspension together with stronger confinement effects: larger intruders leave less space for the surrounding suspension to deform and dilate, which likely increases the resistance to motion. This interpretation is consistent with earlier results by von Kann *et al.*,^51^ who reported lower sinking velocities for intruders of the same size in smaller containers, highlighting the importance of the ratio between intruder size and container width.

**Fig. 4:**
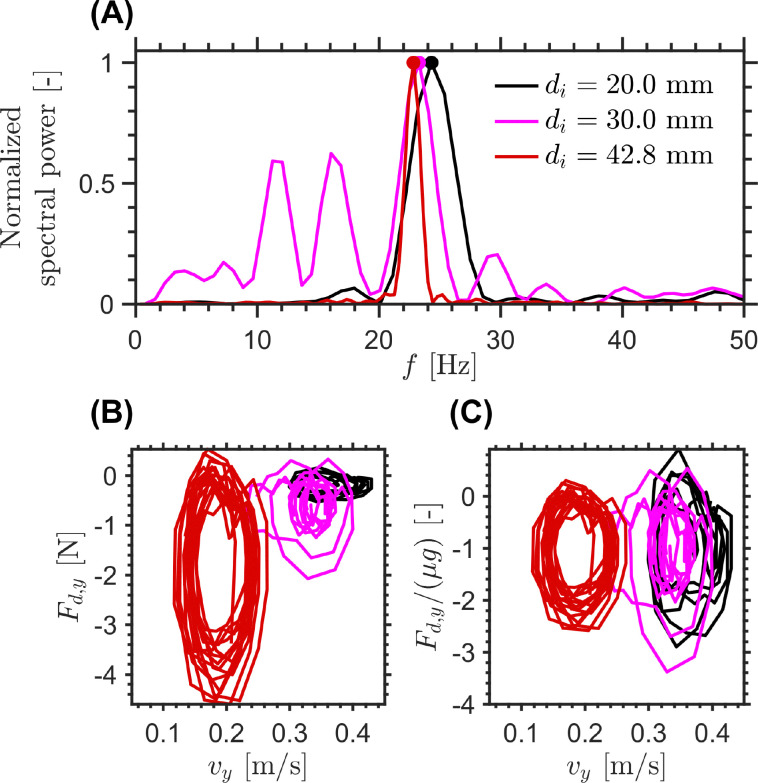
Oscillatory sinking dynamics from the ultra-fast 1D MRI measurements for intruders of diameter *d*_*i*_ = 20.0, 30.0, and 42.8 mm in a cornstarch suspension with solid fraction *ϕ*_0_ = 0.41. (A) Normalized spectral power of the sinking velocity *v*_*y*_, (B) *v*_*y*_*vs. F*_d,*y*_, (C) *v*_*y*_*vs. F*_d,*y*_/(*μg*).

The key observation from the 1D MRI measurements is that, although the mean sinking velocities differ substantially, the oscillatory component of the motion remains of comparable amplitude (≈0.04–0.05 ms^−1^) and frequency for all three intruder sizes. The amplitude is not strictly constant, however, and shows cycle-to-cycle modulation, with approximate values spanning 0.035–0.065 ms^−1^ over the oscillatory sinking window. This point is reinforced by the Fourier spectra shown in Fig. 4A. In each case, the velocity signal exhibits a dominant peak at nearly the same frequency, *i.e.* around 23–25 Hz. The weak dependence of this frequency on *d*_*i*_ suggests that the oscillatory dynamics are not set primarily by the intruder size, but may instead reflect a common characteristic timescale associated with the internal relaxation dynamics of the suspension.

To examine this point more directly, the effective vertical drag force, *F*_d,*y*_, was computed from the measured trajectories using the first sub-equation in eqn (2), and plotted against the instantaneous sinking velocity in Fig. 4B. Following the representation introduced by von Kann *et al.*,^51^ this provides a compact way to visualize the drag response during sinking. In dimensional form, the three datasets occupy different force ranges, as expected from the different buoyancy-corrected masses, *μ*, and mean sinking velocities of the intruders, 〈*v*_*y*_〉. At the same time, all three curves display the same qualitative feature: the drag does not collapse onto a single-valued function of *v*_*y*_, but instead forms skewed hysteretic loops with distinct branches during acceleration and deceleration, indicating that the drag is not determined by the instantaneous velocity alone, but depends on the recent history of the intruder motion.^51^

A more informative comparison is obtained by normalizing the drag force by the buoyancy-corrected weight, *μg*, as shown in Fig. 4C. After this rescaling, the three datasets exhibit loop shapes that are much more alike, even though some asymmetry and skewness remain. Considered together with the similar dominant frequencies in Fig. 4A, this suggests that the oscillatory drag contains a delayed contribution that is not set primarily by the intruder size. Rather than introducing a full constitutive model, we propose a reduced description of the drag in which one part responds instantaneously to the intruder velocity, while another accounts for a finite-time response of the suspension. We therefore write the normalized drag as4
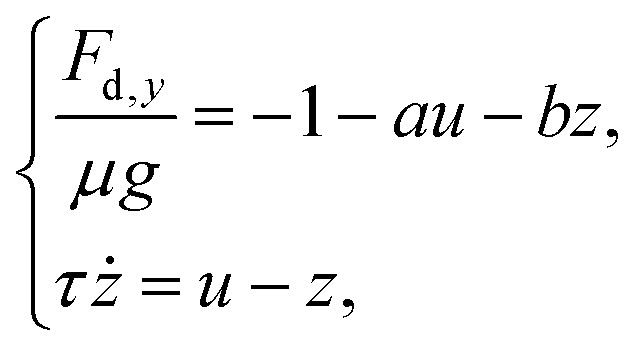
where

5 *u* = *v*_*y*_ − 〈*v*_*y*_〉

is the fluctuation of the sinking velocity about its mean value, the constant term −1 represents the mean drag contribution required to balance the buoyancy-corrected weight *μg*, the term *au* describes the part of the drag that responds directly to the instantaneous velocity fluctuation, whereas the term *bz* accounts for a delayed contribution associated with the finite-time response of the cornstarch suspension.

To represent this delayed response, we introduce an auxiliary variable *z* that relaxes toward the instantaneous velocity fluctuation over a characteristic timescale *τ*. Physically, this delayed term may reflect suspension rearrangements that do not adjust instantaneously to changes in *v*_*y*_. The parameter *b* sets the strength of the delayed contribution, while *τ* characterizes its relaxation time. The coefficients *a* and *b* are therefore treated here as fitted dimensional parameters, without assigning them a direct microscopic interpretation. Since no obvious nondimensionalization emerges based on a characteristic suspension length scale, the reduced-lag description should be regarded as an empirical representation of the measured dynamics rather than as a predictive constitutive model.

**Fig. 5:**
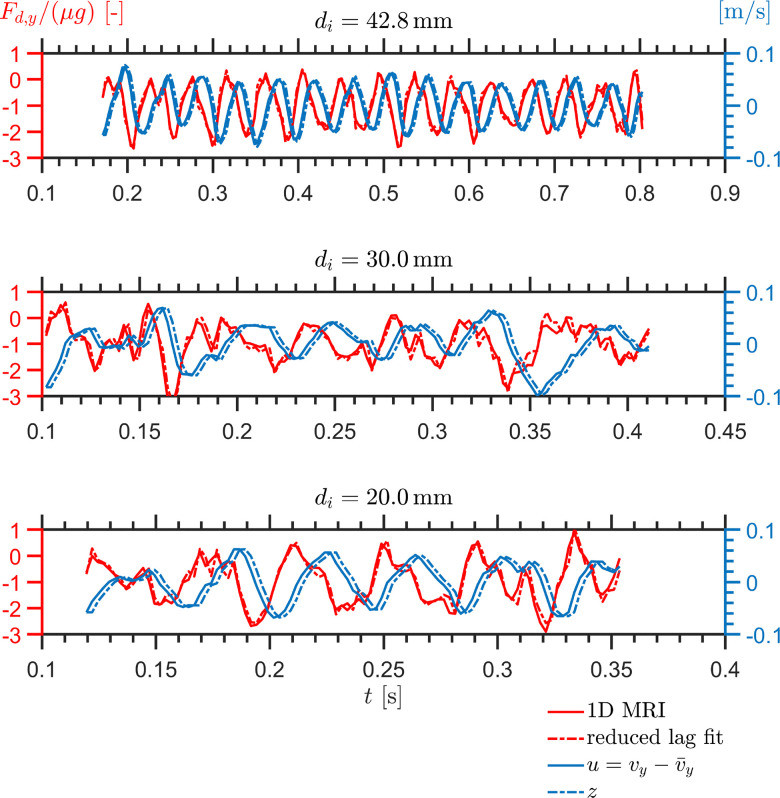
Temporal evolution of the normalized drag force, *F*_d,*y*_/(*μg*), together with the reduced-lag fit (left axis), and of the fluctuation velocities *u* = *v*_*y*_ − *v̄*_*y*_ and *z* (right axis), for intruder diameters *d*_*i*_ = 42.8, 30.0, and 20.0 mm in a cornstarch suspension with solid fraction *ϕ*_0_ = 0.41.

The comparison in Fig. 5 tests the drag decomposition itself. Here, the measured velocity fluctuation *u*(*t*) from the 1D MRI data is used as input to eqn (4), and the corresponding normalized drag is reconstructed. The coefficients (*a*,*b*,*τ*) are determined from the largest intruder and then kept fixed for the medium and small intruders. With this procedure, the reconstructed drag histories reproduce the measured drag reasonably well in all three cases, including the main oscillation period, the phase lag relative to *u*, and a substantial part of the amplitude modulation. At least under the present conditions, this indicates that the measured drag can be decomposed into an instantaneous contribution proportional to *u* and a delayed contribution carried by *z*.

A further step is to combine this reduced drag description with the intruder force balance. Using *mv̇*_*y*_ = *μg* + *F*_d,*y*_ together with eqn (4), and noting that *u̇* = *v̇*_*y*_, gives6
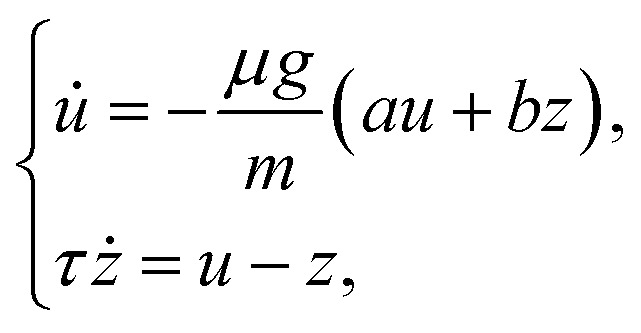
which defines a reduced two-variable dynamical system for the oscillatory part of the sinking motion. In contrast to the earlier phenomenological description of von Kann *et al.*,^51^ this reduced description does not require the oscillation amplitudes or frequencies to be prescribed directly from each experimental trajectory, although it does use the measured mean sinking velocity 〈*v*_*y*_〉 for each case.

For the linear system in eqn (6), the Jacobian has trace7
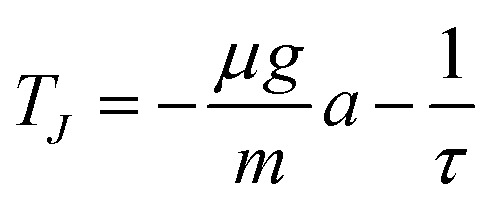
and determinant8
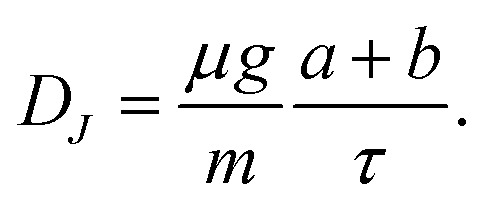


Oscillatory solutions require complex eigenvalues, *i.e. D*_*J*_ > 0 and *T*_*J*_^2^ − 4*D*_*J*_ < 0. In a strictly linear autonomous model, however, oscillations of constant amplitude occur only at the marginal condition *T*_*J*_ = 0. Motivated by the approximately sustained oscillations observed experimentally over the measurement window, we therefore consider the constrained relation9
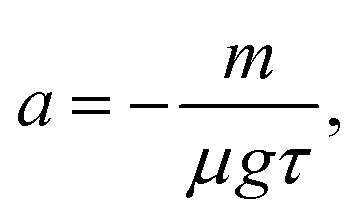
which links the instantaneous drag parameter to the memory time *τ* and places the linear model at marginal oscillatory stability. In this sense, the model should be interpreted as a minimal near-marginal effective description of the measured dynamics, rather than as a complete mechanism for amplitude selection.

**Fig. 6:**
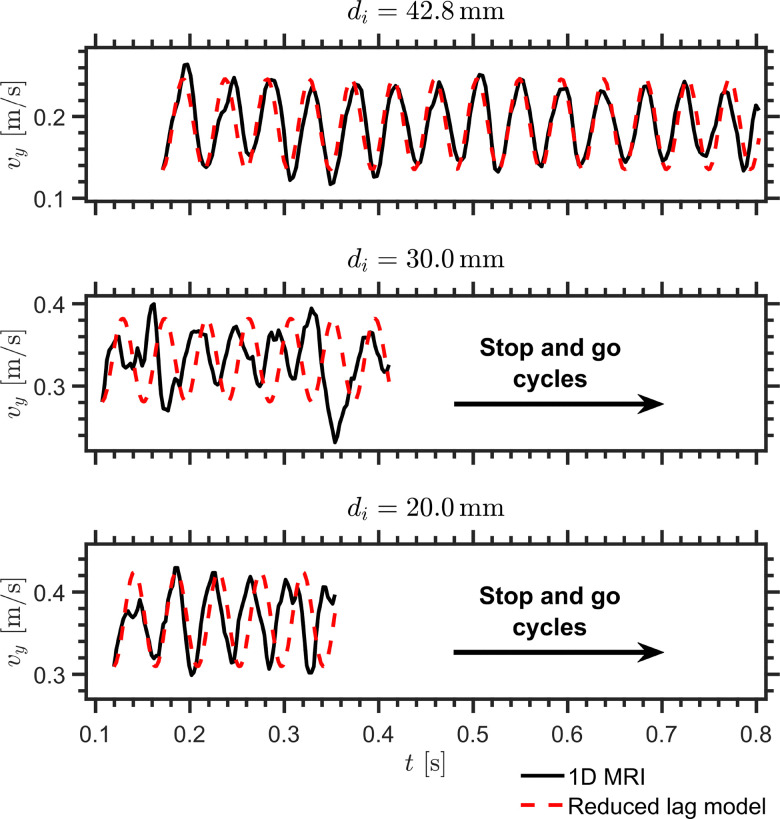
Temporal evolution of the vertical intruder velocity, *v*_*y*_, obtained from the 1D MRI measurements (solid black lines), together with the prediction of the reduced drag-memory model (dashed red lines) using the dimensional parameters *a* = −242.07 s m^−1^, *b* = 243.81 s m^−1^, and *τ* = 6.01 × 10^−4^ s, for intruder diameters *d*_*i*_ = 42.8, 30.0, and 20.0 mm in a cornstarch suspension with solid fraction *ϕ*_0_ = 0.41.

The resulting comparison for the oscillatory sinking velocity is shown in Fig. 6. Using the fitted parameter set *a* = −242.07 s m^−1^, *b* = 243.81 s m^−1^, and *τ* = 6.01 × 10^−4^ s, determined from the largest intruder and then applied unchanged to the other two cases, the reduced linear phenomenological lag model reproduces the oscillation period well and captures the existence of oscillatory sinking in all three cases. The fitted value of *τ* is much shorter than the bulk relaxation times reported in rheometric studies of dense cornstarch suspensions (typically in the range 10^−2^–1 s), suggesting that, within the present model, it is better interpreted as an effective local lag time of the delayed drag contribution than as a macroscopic bulk relaxation time.^55,56^ The agreement is less accurate at the level of the oscillation amplitudes, in particular for the medium-sized intruder, but the model nevertheless captures the dominant oscillation timescale and a substantial part of the observed velocity signal for the presented conditions. This supports the view that delayed drag, rather than intruder size alone, plays an important role in setting the oscillation dynamics over the present parameter range.

We also tested nonlinear extensions of eqn (6) in which a cubic saturation term was added in order to regularize amplitude growth away from the marginal linear point:10
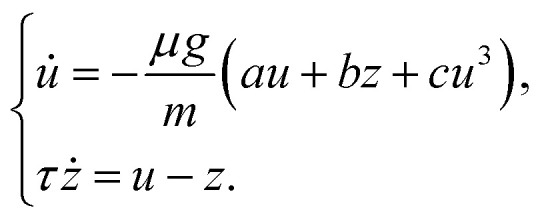


Fitting this system freely to the largest-intruder trajectory produced undamped oscillations with *τ* = 7.34 × 10^−4^ s, *a* = −198.34 s m^−1^, *b* = 200.47 s m^−1^, and *c* = 1 s^3^ m^−3^. The fitted trajectory was nearly indistinguishable from that obtained with the constrained linear model, both for the largest intruder used in the fit and when the same parameter set was applied to the other two intruder sizes. This similarity arises because the fitted parameters compensate each other: in particular, the product *aτ*, which controls the balance between the instantaneous drag response and the memory timescale, remains close to that of the constrained linear fit, while the cubic contribution is small over the observed range of velocity fluctuations. Thus, over the present range of oscillation amplitudes, the cubic term does not substantially improve the description of the measured trajectories or provide a clearer physical interpretation. We therefore do not emphasize it any further. For the present data, the linear near-marginal model appears sufficient as a compact phenomenological summary of the oscillatory sinking regime, while a more detailed constitutive interpretation would require additional evidence.

#### 3.1.2 Suspension response

Having characterized the main features of the intruder motion, we now turn to the accompanying response of the surrounding suspension as revealed by the MRI signal intensity. Beyond tracking the intruder trajectory, the 1D MRI profiles provide qualitative information on spatiotemporal variations of the suspension signal during the oscillatory sinking regime.

The signal intensity maps shown in Fig. 3A–C reveal that the oscillations in the sinking velocity of the intruder are accompanied by nearly vertical striations of reduced MRI signal in the suspension, both above and below the intruder. Similar striations are also visible within the projected region occupied by the intruder, as highlighted in Fig. 3F, which shows the same measurement as Fig. 3C with enhanced contrast. Since the intruder itself is MRI-invisible, these features must arise from signal contributions of the surrounding suspension located beside the intruder within the same horizontal slice. The observed intensity variations therefore indicate that the suspension signal is modulated not only above and below the intruder, but also in its lateral vicinity.

At present, we interpret these signal variations cautiously. Because the magnitude signal in the 1D T1FFE/FLASH acquisition used here can be influenced by factors in addition to liquid content, including flow-induced and microstructural effects, we do not treat the signal changes as a direct quantitative measurement of local liquid fraction. As described in Section 2, the 1D T1FFE/FLASH sequence used here is a time-efficient short-repetition-time gradient-echo acquisition, for which the measured magnitude signal can be affected by motion of the suspension within the excited slice. This is consistent with previous MRI work using the same MRI system and receiver array on MRI-active dry granular materials, which showed that coherent and incoherent particle motion can modulate the MR signal and produce signal enhancements and attenuations, respectively.^53^ It is also supported by previous work on cornstarch suspensions showing that densification during jamming-front propagation can remain very small and even below experimental resolution under impact conditions.^15,48^ We therefore regard the MRI signal variations as qualitative signatures of an evolving suspension state during sinking, most likely associated with suspension motion, microstructural rearrangements, localized deformation, and stress redistribution.

For the largest intruder (Fig. 3E), similar fluctuations in the suspension-related MRI signal are also observed near the bottom of the container. A similar type of response was reported by Waitukaitis and Jaeger,^13^ who identified a dynamic jamming front propagating from the bottom of the intruder toward the bottom of the container after impact. In their study of impactor dynamics, the primary response was caused by the initial impact itself, whereas a secondary bounce-back effect occurred only after the jamming front reached the bottom boundary, at which point the resulting reaction was transmitted back to the intruder almost instantaneously. In the present MRI data, a qualitatively similar picture is suggested by the bottom signal oscillations, which weaken over time but remain broadly consistent in frequency with the oscillations in the sinking velocity of the intruder. Moreover, the inclination of the first nearly vertical striation, associated with the initial impact of the largest intruder, yields an estimated propagation speed of approximately *v*_f_ ≈ 3.46 ms^−1^. For comparison, using eqn (1) with the estimated impact velocity and *ϕ*_0_ = 0.41 gives *v*_f_ ≈ 3.49 ms^−1^ for *ϕ*_*J*_ = 0.57. This value of *ϕ*_*J*_ is close to the range of jamming volume fractions reported for different batches of cornstarch suspensions in the literature, *ϕ*_*J*_ = 0.51–0.56,^15,29,45^ supporting the interpretation of the first striation as a dynamic jamming front. The lower slopes of the subsequent striations suggest that later propagating disturbances travel at lower speeds.

**Fig. 7:**
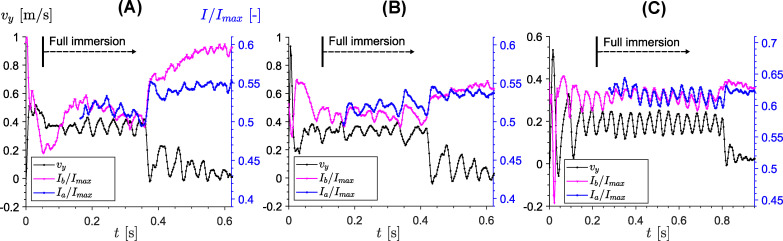
Temporal evolution of the sinking velocity of the intruder and the signal intensity, averaged over the estimated affected regions above (*I*_a_/*I*_max_) and below (*I*_b_/*I*_max_) the intruder, extending from its surface to approximately 5–10 mm (depending on size) into the suspension. Panels (A)–(C) correspond to intruder diameters of *d*_*i*_ = 20, 30, and 42.8 mm, respectively, for a cornstarch suspension with solid fraction *ϕ*_0_ = 0.41.

Fig. 7 shows the temporal evolution of the sinking velocity (black lines) together with normalized MRI signal intensities averaged over the affected regions identified from the signal maps, both below and above the intruder. The corresponding characteristic extent of these affected regions is approximately 5 mm for the large intruder, 8.5 mm for the medium intruder, and 10 mm for the small intruder, increasing in the same order as the mean sinking velocity. These characteristic lengths should be regarded as approximate, because the lower boundary of the low-intensity region is not sharply defined and depends to some extent on the threshold used to identify recovery toward the undisturbed MRI signal (in this case ≈95%), which itself contains noise. We therefore do not interpret them as the thickness of a uniquely defined jammed layer. Rather, they provide an operational estimate of the vertical extent over which the suspension state is measurably perturbed beneath the intruder.

The plots reveal a clear temporal correlation between the intruder motion and the suspension response. In all three cases, the MRI signal measured within the affected regions above and below the intruder oscillates with the same characteristic frequency as the sinking velocity. Within the temporal resolution of the measurements, the extrema of the intensity traces do not show a clear systematic lag relative to those of *v*_*y*_. At the level of the present data, the oscillatory sinking is thus accompanied by repeated growth and partial relaxation of an affected region around and beneath the intruder. In a dynamic-jamming picture, this behavior is consistent with intermittent dilation-relaxation cycles near a jamming threshold: as the intruder moves, the surrounding suspension develops an extended perturbed region associated with evolving contact networks and stress transmission, which then relaxes again as the motion slows. The stop and go cycles, on the other hand, are likely to result from stronger transient shear-jammed states developing beneath the intruder due to increasing confinement (the mechanism described by von Kann *et al.*^51^).

### 3.2 Insights from 2D MR velocity measurements

In addition to the 1D MRI measurements, 2D phase-contrast MR velocimetry was performed in order to resolve the motion of both the intruder and the surrounding suspension in the imaging plane. These experiments were carried out at a slightly higher stress-free solid fraction, *ϕ*_0_ = 0.44, than the 1D MRI measurements (*ϕ*_0_ = 0.41), in order to slow the dynamics sufficiently to remain within the temporal resolution of the 2D velocimetry sequence (Δ*t* = 62 ms). The results shown here correspond to the largest intruder, with diameter *d*_*i*_ = 42.8 mm.

**Fig. 8:**
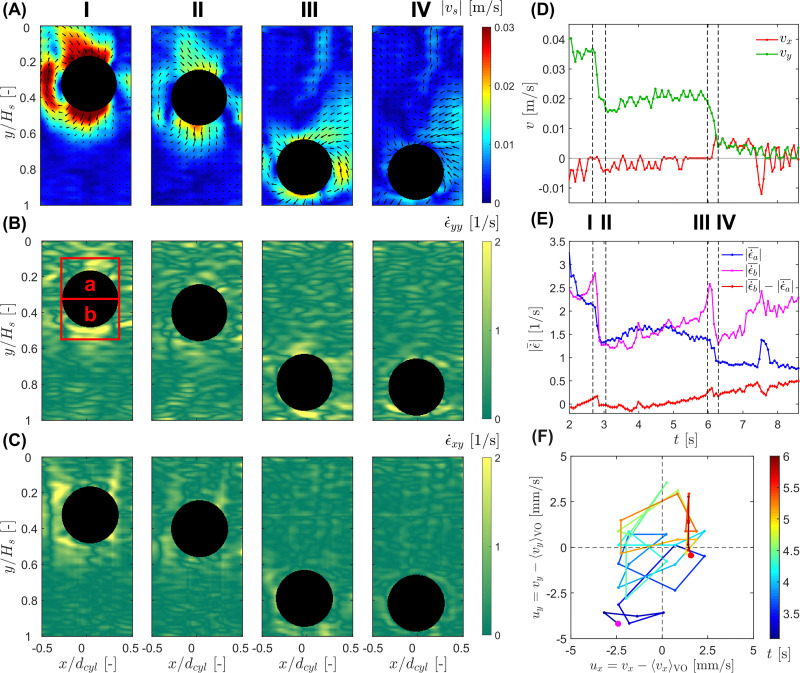
Visualization of the 2D phase-contrast MR velocimetry and strain rate measurements for an intruder of diameter 42.8 mm sinking in a cornstarch suspension with solid fraction *ϕ*_0_ = 0.44. Panel (A) shows the magnitude of the in-plane suspension velocity, |*v*_s_|, with arrows indicating the flow direction. Panels (B) and (C) show the two dominant in-plane strain rate tensor components, *ε̇*_*yy*_ and *ε̇*_*xy*_, respectively, at selected times *t*_I_ = 2.6 s, *t*_II_ = 3.1 s, *t*_III_ = 6.0 s, and *t*_IV_ = 6.3 s. Panel (D) shows the horizontal and vertical intruder velocities, *v*_*x*_ and *v*_*y*_, as functions of time. Panel (E) shows the spatially averaged strain rate magnitudes above and below the intruder, 
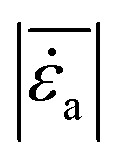
 and 
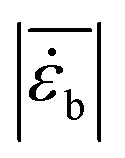
, obtained over rectangular regions of width equal to the intruder diameter and height equal to 0.75*d*_*i*_, together with the corresponding strain rate asymmetry, 
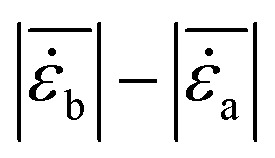
, shown in red. Panel (F) shows the horizontal velocity fluctuation, *u*_*x*_ = *v*_*x*_ − 〈*v*_*x*_〉_VO_, plotted against the vertical velocity fluctuation, *u*_*y*_ = *v*_*y*_ − 〈*v*_*y*_〉_VO_, during the bulk velocity-oscillation window. The regions used for the averaging are indicated by the red rectangles in panel (B).

Fig. 8 presents the measured 2D suspension velocity field and the associated in-plane strain rate field around the sinking intruder. The magnitude of the in-plane suspension velocity is defined as 
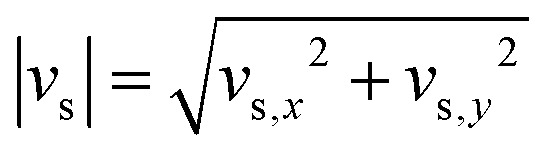
 (shown in panel A), while the strain rate magnitude is computed as11
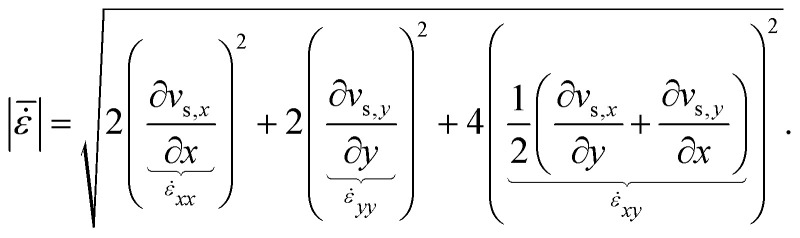


Here, *v*_s,*x*_ and *v*_s,*y*_ denote the suspension velocities in the *x* and *y* directions, while *ε̇*_*xx*_, *ε̇*_*yy*_ (panel B), and *ε̇*_*xy*_ (panel C) are the corresponding in-plane strain rate tensor components.^15^ The quantity 
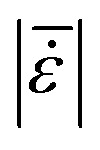
 was obtained by central differencing and then spatially averaged over two rectangular regions, one above and one below the intruder centroid (spanning 0.75*d*_*i*_ from it), yielding 
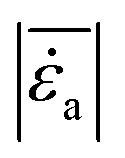
 and 
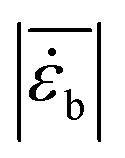
, respectively.

Three dynamical stages can be distinguished from Fig. 8D: an impact transient (2.0 s < *t* < 2.7 s), an intermediate oscillatory sinking regime (3.1 s < *t* < 6.0 s), and a stop and go regime as the intruder approaches the bottom boundary (6.3 s < *t* < 8.6 s). In the oscillatory sinking regime, the mean vertical velocity is approximately *v*_*y*_ ≈ 0.02 ms^−1^, while both *v*_*y*_ and *v*_*x*_ exhibit oscillations with a characteristic frequency of approximately 4.3 Hz and amplitudes of order 2.5 mm s^−1^. The presence of oscillations in both velocity components is one of the most distinctive outcomes of the 2D MRI measurements. It shows that the motion is not purely one-dimensional, but involves lateral velocity oscillations of the intruder as it sinks. This point is further illustrated in Fig. 8F, where the velocity fluctuations *u*_*x*_ = *v*_*x*_ − 〈*v*_*x*_〉_VO_ and *u*_*y*_ = *v*_*y*_ − 〈*v*_*y*_〉_VO_ are plotted against each other during the steady oscillatory sinking window. The trajectory does not collapse onto a single line or form a simple closed ellipse, indicating that the horizontal and vertical fluctuations are not well described by a fixed phase relation or by a simple harmonic mode. Instead, the comparable magnitudes of *u*_*x*_ and *u*_*y*_ suggest a laterally unsteady center-of-mass motion of the intruder, likely associated with asymmetric and time-dependent stress transmission around the intruder.

The 2D velocity and strain rate fields (panels A, B, C and E) further show that the suspension response evolves differently above and below the intruder. During the early impact stage, the averaged strain rate magnitude above the intruder, 
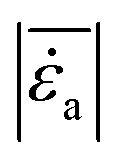
, initially exceeds that below, 
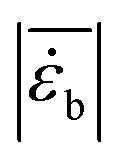
. As the impact response relaxes, however, this balance reverses: 
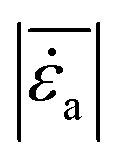
 decreases while 
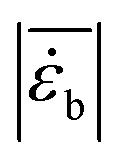
 rises and reaches a local maximum (≈2.8 s^−1^) near *t* ≈ 2.7 s, coincident with the strong deceleration visible in Fig. 8D. Over the subsequent interval, both quantities decrease rapidly toward lower values (≈1.5 s^−1^) as the system enters the slower oscillatory sinking regime. A similar tendency is observed again later in the experiment. During the oscillatory sinking regime, the strain rate magnitude above the intruder, 
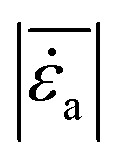
, decreases while the strain rate magnitude below the intruder, 
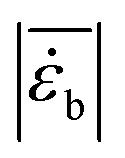
, increases and remains larger, reaching a second maximum (≈2.5 s^−1^) near *t* ≈ 6.0 s just before the onset of arrest. The strain rate asymmetry shown in red in Fig. 8E makes this trend particularly clear: the deformation field becomes progressively more concentrated beneath the intruder as the motion slows and the system approaches the stop and go regime.

**Fig. 9:**
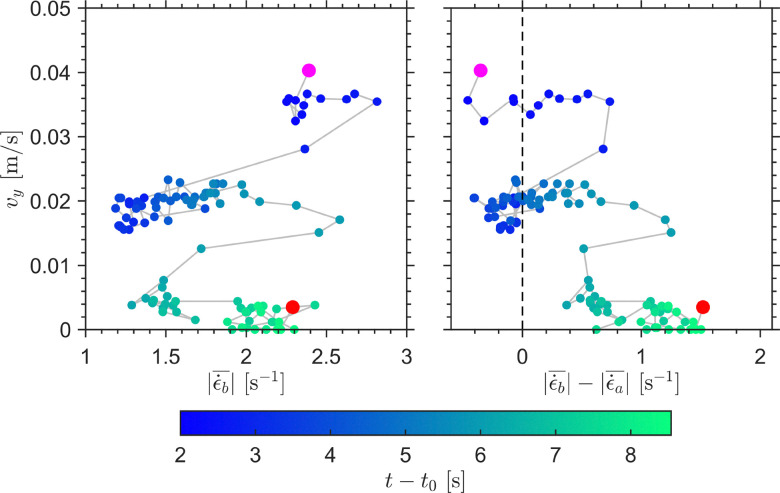
Hysteresis plots of the intruder sinking velocity, *v*_*y*_, shown as a function of 
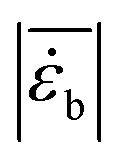
 (left), and of the strain rate magnitude difference 
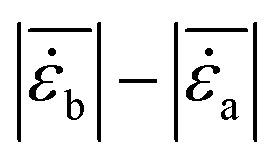
 between the regions below and above the intruder (right) for an intruder of diameter *d*_*i*_ = 42.8 mm in a cornstarch suspension with solid fraction *ϕ*_0_ = 0.44. The color scale indicates time progression through the measurement window.

The hysteresis plots in Fig. 9 provide a compact view of the coupling between intruder motion and the evolving deformation field. In neither the 
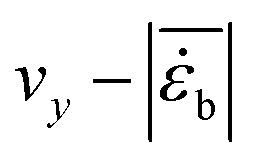
 plane nor the 
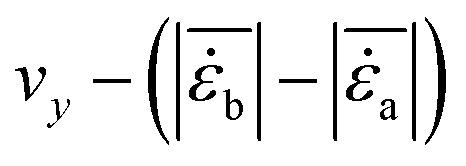
 plane do the data collapse onto a single curve. Instead, the trajectory separates into distinct branches corresponding to the impact transient, the oscillatory sinking regime, and the stop and go cycles. This non-single-valued behavior shows that the intruder velocity is not controlled by an instantaneous local deformation measure alone, but depends on the evolving, spatially heterogeneous state of the suspension. In contrast to this pronounced hysteresis, an analogous drag analysis for the 2D MRI measurements at *ϕ*_0_ = 0.44, performed in the same spirit as for the 1D MRI experiments at *ϕ*_0_ = 0.41, revealed only a very weak hysteretic signature. There, the vertical drag remained close to the buoyancy-corrected weight contribution, *F*_d,*y*_ ≈ −*μg*, with oscillatory deviations of less than about 1% (corresponding to hysteretic variations of approximately 0.01 N around the mean value 〈*F*_d,*y*_〉 ≈ −1.78 N during all sinking regimes). This much weaker hysteresis observed in the higher-solid-fraction measurement suggests that the suspension forms a more robust load-bearing structure, making the drag response less sensitive to repeated restructuring.

Taken together, Fig. 8 and 9 show that the transition toward arrest is accompanied by an increasing asymmetry of the deformation field, with progressively stronger deformation below the intruder than above it. Combined with the observed horizontal oscillations of the intruder, this points to a laterally unsteady and mechanically heterogeneous suspension response. In this sense, the 2D MRI measurements are broadly consistent with the 1D interpretation of repeated growth and partial relaxation of a perturbed region around and beneath the intruder, while additionally suggesting that this region becomes increasingly asymmetric and concentrated below the sphere as arrest is approached. Consistent with this picture, the stop and go cycles may reflect stronger transient shear-jammed states developing preferentially beneath the intruder as confinement increases near the bottom boundary.

## 4 Conclusion

Ultra-fast magnetic resonance imaging (MRI) was employed to investigate the sinking of spherical intruders of identical density but different diameters in cornstarch-water suspensions. The 1D MRI measurements at *ϕ*_0_ = 0.41 provided non-invasive access to the vertical intruder motion together with qualitative information on the evolving suspension signal, while complementary 2D phase-contrast MRI measurements at *ϕ*_0_ = 0.44 resolved the in-plane motion of both the intruder and the surrounding suspension for the largest intruder. Across the 1D measurements, the sinking motion proceeded through three distinct regimes, namely an impact transient, an oscillatory quasi-steady sinking regime, and stop and go cycles near the bottom boundary. Larger intruders were found to sink more slowly, while the oscillations in the intermediate sinking regime exhibited similar characteristic amplitudes and frequencies for all three intruder sizes. A reduced drag-memory model, in which the memory term may phenomenologically represent delayed particle rearrangements and frictional contact dynamics, further showed that these oscillatory velocities can be described reasonably well using a single fitted parameter set across the three intruder sizes, supporting the idea that the drag on the intruder depends not only on its instantaneous velocity but also on the recent history of the surrounding suspension.

The 1D MRI signal maps further revealed that the oscillations in intruder velocity are accompanied by oscillatory modulations of the MRI signal both above and below the intruder, and also in its lateral vicinity. Because the MRI signal in this system can be influenced by flow, relaxation, and microstructural effects in addition to liquid content, these variations were not interpreted as direct quantitative measurements of local solid fraction. Instead, they were taken as qualitative signatures of an evolving perturbed region around the intruder that repeatedly grows and partially relaxes during oscillatory sinking. Within a dynamic-jamming picture, this behavior is consistent with intermittent dilation-relaxation cycles near a jamming threshold.

Complementary 2D phase-contrast MRI measurements at *ϕ*_0_ = 0.44 showed that, in addition to the vertical oscillations, oscillations in the horizontal intruder velocity are also present, with a comparable characteristic frequency and amplitude. This indicates that the oscillatory sinking is not purely one-dimensional, but involves lateral motion together with an asymmetric and time-dependent suspension response in the imaging plane. The 2D velocity and strain rate fields further revealed clear heterogeneity between the regions above and below the intruder, with deformation becoming progressively more concentrated beneath the intruder as the motion slowed and approached arrest. At the same time, the much weaker drag hysteresis observed at *ϕ*_0_ = 0.44 compared with the 1D measurements at *ϕ*_0_ = 0.41 suggests that, at the higher solid fraction, the suspension remains closer to a persistently stress-bearing frictional state, so that the mean drag stays near the buoyancy-corrected weight.

Taken together, these results show that intruder sinking in concentrated cornstarch suspensions is strongly history-dependent and accompanied by a spatially heterogeneous suspension response that evolves with confinement and proximity to arrest. More broadly, the MRI measurements demonstrate how non-invasive imaging can connect intruder kinematics to the evolving state of the surrounding opaque suspension, thereby providing experimental constraints for reduced models of delayed drag and for future constitutive descriptions of oscillatory sinking and transient jamming.

Future work could build on these results by systematically varying the container geometry in order to quantify confinement and wall effects, and by extending the velocimetry to fully three-dimensional MRI measurements. In addition, dedicated MRI sequence comparisons, for example varying the spoiling scheme, flow compensation, and excitation parameters, could help decouple suspension-motion-induced signal changes from variations associated with liquid content or microstructural rearrangements. It would also be valuable to develop models that explicitly incorporate spatial heterogeneity and memory effects, and to compare such models with numerical simulations, for example using CFD-DEM or related approaches informed and validated by the MRI data. Such combined experimental and computational studies could help relate the measured macroscopic dynamics to the underlying evolution of contact networks, local rearrangements, and force transmission in shear thickening suspensions.

## Author contributions

Nikolay K. Kirov: experimental setup design, experiments, formal analysis (including code writing, data curation, visualization), writing – original draft, writing – review and editing. Christopher P. McLaren: experiments, supervision, writing – review and editing. Klaas P. Pruessmann: funding acquisition, resources, writing – review and editing. Christoph R. Müller: funding acquisition, resources, conceptualization, supervision, writing – review and editing. Alexander Penn: conceptualization, experimental setup design, experiments, supervision, writing – review and editing.

## Conflicts of interest

The authors declare that they have no known competing financial interests or personal relationships that could have appeared to influence the work reported in this paper.

## Data Availability

The MRI data supporting this article are available at https://doi.org/10.5281/zenodo.20288229. The experimental procedures and data analysis methods are described in the article. Additional analysis files not included in the repository can be made available by the corresponding author upon reasonable request.
